# 

**Published:** 1883-06

**Authors:** 


### SOUTHERN DENTAL ASSOCIATION.

Dr. L. D. Carpenter, President, announces that the time of meeting of this Association has been changed to Tuesday, July, 31st, 1883, in Atlanta, Georgia. Dr. D. Hopps, President of the Georgia State Dental Society, also announces that their meeting will be held on Monday, July 30th 1883, at the same place,

The change of time has been made to accommodate many

Southern dentists who wish to attend the meetings of the American and other Associations in August.

---

### PENNSYLVANIA STATE DENTAL SOCIETY.

The Pennsylvania State Dental Society will convene at Cresson Springs on the main line of the P. R. R., July 31st, at 10 A. M., and contiuue in session four days.

For all informatfon address.

W. H. FUNDENBERG,
*Cor. Secretary.*

330 Penn Avenue.
Pittsburgh, Pa.

---

### EDITORIAL, ETC.

### To Readers and Correspondents.

Communications intended for publication, and exchange journals and papers, should be addressed to the EDITOR OF THE AMERICAN JOURNAL OF DENTAL SCIENCE, 259 N. Eutaw St., Baltimore, Md. All letters relating to the business of the journal, or containing cash remittances, to be directed to Messrs. SNOWDEN & COWMAN. Articles for publication should be received by the editor at least three weeks previous to the first of the month on which the number in which it is designed for them to appear, is issued.

EDITORIAL, ETC.

The Composition and Effects of Chloral Hydrate....... 89

MONTHLY SUMMARY.

The Care of the Teeth....... 91
Under the Surgeon's Touch....... 93
A Rare Case of Exostosis....... 94
Foreign Bodies in the Air Passages....... 94
Hardening Plaster Casts ....... 95
Our Dental Brethern....... 95
A Simple Method of Producing Local Anæsthesia....... 96
Dental Formation in the Nasal Cavity....... 96

☞ The American Journal of Dental Science will contain fifty pages of reading matter in each number, making a volume of about

*Six Hundred* pages,

EXCLUSIVE OF ADVERTISEMENTS.

THE

### AMERICAN JOURNAL

OF

### DENTAL SCIENCE.

The *Journal* is issued on the first of each month and contains not less than *fifty* pages of reading matter in each number, exclusive of advertisements, making a volume of *six hundred* pages per annum.

In each number will be found Original Communications, Correspondence, Selected Articles, Monthly Summary, Bibliographical Notices and Editorial Matter, and every effort will be exerted to render the *Journal* worthy of the patronage of the Dental profession.

A generous co-operation is respectfully solicited from the members of the profession; and as the *Journal* has now a printing office of its own, which materially lessens the cost of its publication, it will b issued at the reduced subscription price of

**$2.50 Per Annum in Advance.**

Communications are respectfully solicited on all subjects pertaining to the practice of dentistry, as well as reports of cases occurring in dental practice.

### RATES OF ADVERTISING.

| | 1 MO. | 2 MO. | 3 MO. | 6 MO. | 12 MO. |
|---|---|---|---|---|---|
| 1 PAGE. | $15 00 | $22 50 | $30 00 | $50 00 | $100 00 |
| ½ " | 10 00 | 15 00 | 20 00 | 30 00 | 50 00 |
| ¼ " | 7 00 | 11 00 | 15 00 | 20 00 | 30 00 |
| ⅛ " | 5 00 | 8 00 | 11 00 | 15 00 | 20 00 |

All original communications designed for publication, works for review, new instruments for notice, exchanges, &c., should be directed to the editor, 259 N. Eutaw St., Baltimore, Md.

All advertisements and subscriptions should be addressed to

SNOWDEN & COWMAN,
*Publishers of the Am. Journal of Dental Science.*
86 W FAYETTE ST. BET. CHARLES & LIBERTY STS.,
BALTIMORE, MD

